# Convergent Evidence from Multimodal Imaging Reveals Amygdala Abnormalities in Schizophrenic Patients and Their First-Degree Relatives

**DOI:** 10.1371/journal.pone.0028794

**Published:** 2011-12-08

**Authors:** Lin Tian, Chun Meng, Hao Yan, Qiang Zhao, Qi Liu, Jun Yan, Yonghua Han, Huishu Yuan, Lifang Wang, Weihua Yue, Yanbo Zhang, Xinmin Li, Chaozhe Zhu, Yong He, Dai Zhang

**Affiliations:** 1 Institute of Mental Health, Peking University, Beijing, China; 2 Key Laboratory for Mental Health, Ministry of Health, Beijing, China; 3 State Key Laboratory of Cognitive Neuroscience and Learning, Beijing Normal University, Beijing, China; 4 Department of Radiology, The Third Hospital, Peking University, Beijing, China; 5 Department of Psychiatry, Faculty of Medicine, University of Manitoba, Winnipeg, Canada; Institute of Automation, Chinese Academy of Sciences, China

## Abstract

**Background:**

Shared neuropathological features between schizophrenic patients and their first-degree relatives have potential as indicators of genetic vulnerability to schizophrenia. We sought to explore genetic influences on brain morphology and function in schizophrenic patients and their relatives.

**Methods:**

Using a multimodal imaging strategy, we studied 33 schizophrenic patients, 55 of their unaffected parents, 30 healthy controls for patients, and 29 healthy controls for parents with voxel-based morphometry of structural MRI scans and functional connectivity analysis of resting-state functional MRI data.

**Results:**

Schizophrenic patients showed widespread gray matter reductions in the bilateral frontal cortices, bilateral insulae, bilateral occipital cortices, left amygdala and right thalamus, whereas their parents showed more localized reductions in the left amygdala, left thalamus and right orbitofrontal cortex. Patients and their parents shared gray matter loss in the left amygdala. Further investigation of the resting-state functional connectivity of the amygdala in the patients showed abnormal functional connectivity with the bilateral orbitofrontal cortices, bilateral precunei, bilateral dorsolateral frontal cortices and right insula. Their parents showed slightly less, but similar changes in the pattern in the amygdala connectivity. Co-occurrences of abnormal connectivity of the left amygdala with the left orbitofrontal cortex, right dorsolateral frontal cortex and right precuneus were observed in schizophrenic patients and their parents.

**Conclusions:**

Our findings suggest a potential genetic influence on structural and functional abnormalities of the amygdala in schizophrenia. Such information could help future efforts to identify the endophenotypes that characterize the complex disorder of schizophrenia.

## Introduction

Schizophrenia, which affects approximately 1% of the population worldwide, is a common and complex mental disorder characterized by psychosis, social withdrawal, cognitive deficit and emotional impairment [1], [2]. The rate of schizophrenia is higher in the relatives of patients than in the general population. A great number of epidemiological evidences collected from adoption, twin and family studies have shown that this risk is genetic, with a nearly tenfold increase in risk associated with the occurrence of an affected first-degree relative [3], [4].

Since the advent of in vivo neuroimaging techniques, the presence of structural abnormalities and functional impairments has been well established in brains with schizophrenia [5], [6]. Convergent evidence has identified replicable structural and functional abnormalities of the frontal lobes, superior temporal gyrus, amygdala, hippocampus and thalamus in schizophrenia [7], [8], [9], [10], [11], [12]. Even unaffected first-degree relatives of schizophrenic patients show a similar but milder pattern of the brain abnormalities observed in schizophrenia, such as reduced hippocampal volume and hypoactivity of the amygdala during sadness [13], [14]. These similar neuroimaging abnormalities in unaffected relatives are likely to be manifestations of the genes that predispose people to schizophrenia [15], [16]. Hence, the study of unaffected first-degree relatives can be a complementary approach for seeking biological markers associated with genetic risk (endophenotypes) and may help bridge the gap between vulnerability genes and the clinical syndrome [17], [18], [19]. A recent conceptualization of endophenotypes has proposed the term ‘extended-endophenotypes’ for a functionally linked set of endophenotypes to elucidate the pathophysiological pathways in schizophrenia [20]. However, few family studies have focused on whether finding structural and functional imaging features connected with the same, specific brain regions could imply that these combinations of features in this specific brain region have a genetic basis. Consistent findings of co-occurrences of abnormalities in the structure and function of brain regions in schizophrenic patients as well as in their first-degree relatives could shed additional light on this topic.

The idea that dysconnection is the core pathophysiology of schizophrenia has become increasingly influential [21], [22], [23] and has been strongly supported by evidence of widespread disturbances in functional connectivity in schizophrenia based on functional neuroimaging techniques in task-dependent as well as on task-independent, i.e., resting state studies [24], [25], [26]. Because a large number of studies have indicated brain structural alterations in schizophrenia, connecting brain structure with function can offer valuable insights into the pathology of schizophrenia [27], [28], [29]. Structural MRI studies have revealed that genetic factors may play a major part in brain structural abnormalities in schizophrenia [30], [31]. In addition, previous work suggested that resting-state functional connectivity (RSFC) is under genetic control and that the disturbed patterns were quite similar in schizophrenic patients and their first-degree relatives [15], [32], [33]. Therefore, inferring that schizophrenic patients and their first-degree relatives share some neuropathological features of their brain morphology and resting-state brain function is reasonable. Using a multimodal imaging strategy, we studied schizophrenic patients (SZ, n = 33) and their unaffected biological parents (PA, n = 55) to test the hypothesis that altered RSFC may accompany structural abnormalities in schizophrenic patients and that a similarly changed pattern may appear in their unaffected parents.

## Results

### Voxel-based Morphometry Analysis

**Figure 1:**
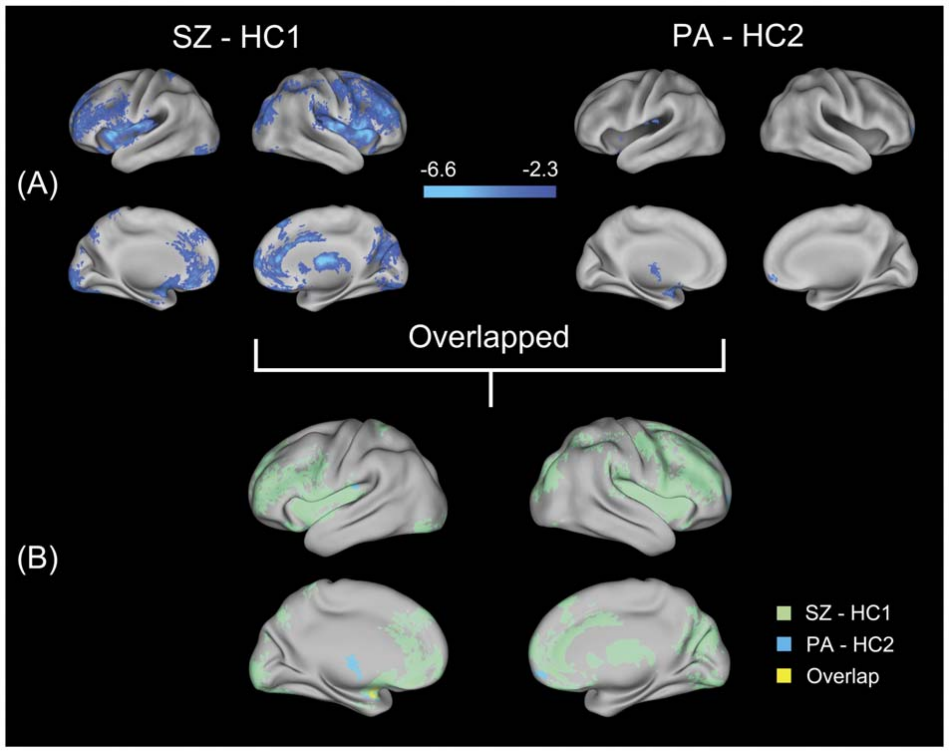
Surface renderings representing the results from the analysis of structural MRI data. Statistical parametric maps of VBM analysis are superimposed on the population, landmarked-and surface-based (PALS) atlas of human cerebral cortex [93] using CARET software (http://brainvis.wustl.edu). (A) Regions with significantly (*p*<0.05, corrected) decreased gray matter density in schizophrenic patients and their parents compared with their respective counterparts are shown in cool color. Color bar indicates the *z*-value. (B) An overlapping surface rendering of the between-group statistical parametric maps (SZ versus HC1, light green; PA versus HC2, light blue). Yellow areas represent areas of overlap between the two statistical parametric maps. SZ, schizophrenic patients; HC1, healthy controls for schizophrenic patients; PA, parents of schizophrenic patients; HC2, healthy controls for parents.

To identify gray matter alternations in schizophrenia families, we performed a voxel-based morphometry (VBM) analysis. Compared with their counterpart healthy controls (HC1, n = 30), the schizophrenia group showed significantly decreased gray matter density (GMD) primarily in the bilateral orbitofrontal cortices (OFC), bilateral dorsolateral prefrontal cortices (DLPFC), bilateral anterior cingulate cortices (ACC), bilateral precunei, bilateral insular cortices, bilateral superior parietal cortices, bilateral lingual cortices, left cerebellum, left amygdala, left parahippocampus (extended to left hippocampus), right middle occipital gyrus and right thalamus (Figure 1A, Table S1). Compared with HC1, no significant increases in GMD were found in the schizophrenia patients. Relative to their counterpart healthy controls (HC2, n = 29), the parent group showed significantly decreased GMD mainly in the left thalamus, left insula, left amygdala and right OFC (Figure 1A, Table S2). Also, no significant increases in GMD appeared in the parent group compared with HC2. In addition, the SZ and PA groups both showed GMD changes in the left amygdala (cluster-size = 101 mm^3^, geometric center: [−28, 4, −16] in Montreal Neurological Institute (MNI) space) (Figure 1B).

### Functional Connectivity Analysis

We were also interested in whether the patients and their parents would show similar abnormalities in RSFC of the amygdala. Although the VBM analysis revealed no common GMD changes between the schizophrenia and their parents in the right amygdala, we still concerned its functional integration during the resting state. To this end, we performed the RSFC analyses using the bilateral amygdalae as regions of interest (ROIs).

### Within-Group RSFC Patterns

**Figure 2:**
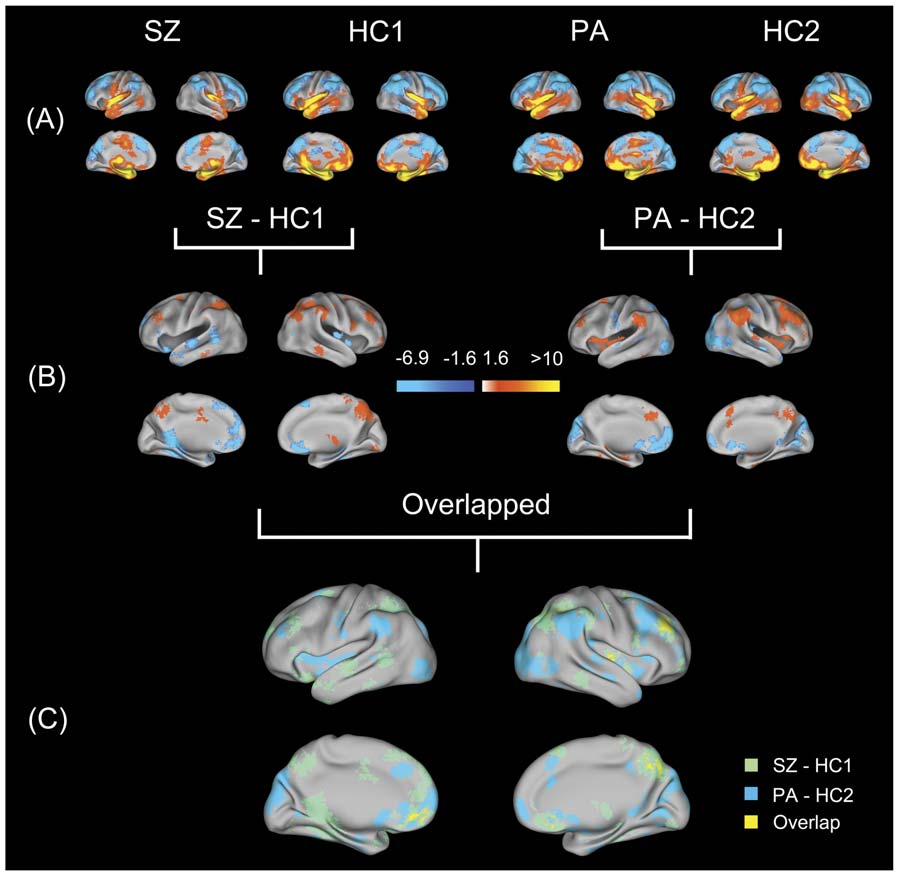
Surface renderings representing the results from the analysis of functional MRI data. Statistical parametric maps of functional connectivity analysis of the left amygdala are superimposed on the population, landmarked-and surface-based (PALS) atlas of the human cerebral cortex [93] using CARET software (http://brainvis.wustl.edu). (A) Within-group functional connectivity patterns. Cool color and warm color, respectively, are used to indicate regions with significantly negative connectivity and significantly positive connectivity (*p*<0.05, corrected) with the left amygdala. (B) Between-group functional connectivity patterns. Regions with significantly (*p*<0.05, corrected) altered functional connectivity (compared with their representative counterparts) with the left amygdala in schizophrenic patients and their parents are shown. Color bar indicates the *z*-value. (C) An overlapping surface rendering of the between-group statistical parametric maps (SZ versus HC1, light green; PA versus HC2, light blue). Yellow areas represent areas of overlap between the two statistical parametric maps. SZ, schizophrenic patients; HC1, healthy controls for schizophrenic patients; PA, parents of schizophrenic patients; HC2, healthy controls for parents.

One-sample *t*-tests revealed similar patterns of functional connectivity (FC) for the left and right amygdalae within each group (Figure 2A, Figure S1A). In the healthy controls, the bilateral amygdalae showed similar patterns of significant functional connectivity including a number of regions, e.g., positive connectivity with the bilateral medial prefrontal cortices (MPFC), bilateral ACCs, bilateral insulae, and bilateral temporal cortices, and negative connectivity with the bilateral superior frontal cortices (SFC), bilateral DLPFCs, bilateral posterior cingulate cortices and the precuneus. Similar patterns were found in the other groups.

### Between-Group RSFC Patterns

#### Left amygdala FC

Relative to their controls, the schizophrenic patients exhibited significantly reduced positive connectivity to the left SFC, left MPFC, left middle temporal gyrus, right hippocampus and bilateral OFCs. This SZ group showed reduced negative connectivity to the right DLPFC, right superior parietal lobule and right angular cortex (Figure 2B, Table S3) and also showed increased positive connectivity to the right caudate nucleus and right globus pallidum. No enhances in negative connectivity were observed in the SZs compared with the HC1 group. Relative to their controls, the PAs showed significantly reduced positive connectivity to the left OFC, left MFG, left cuneus and bilateral caudate nuclei. They also showed reduced negative connectivity to the right DLPFC, right inferior parietal lobule and right precuneus (Figure 2B, Table S4). Enhanced negative connectivity was observed primarily to the bilateral middle occipital gyri in the PAs. In addition, schizophrenic patients and their parents both showed FC changes primarily to the left OFC, right DLPFC and right precuneus (Figure 2C, Table 1).

**Table 1:** Brain regions with functional connectivity changes with the left amygdala in common between schizophrenic patients and their parents.

Regions	Side	BA	Cluster-size (k)^a^	Coordinates of geometric centers^b^	Mean connectivity *z* values			
					SZ	HC1	PA	HC2
*Decreased negative connectivity*								
Middle frontal cortex/Pars triangularis	R	46/45/9	89	38 38 30	−0.11(0.15)^c^ *	−0.24(0.15)*	−0.11(0.15)*	−0.24(0.14)*
Middle frontal/Superior frontal cortex	R	8	29	28 10 56	−0.12(0.14)*	−0.23(0.10)*	−0.10(0.17)*	−0.21(0.16)*
Precuneus	R	7	28	10 −66 48	−0.08(0.20)*	−0.19(0.11)*	−0.12(0.14)*	−0.21(0.11)*
Middle frontal/Superior frontal cortex	L	6/8	17	−27 5 60	−0.05(0.14)	−0.16(0.14)*	−0.10(0.19)*	−0.22(0.21)*
Angular/Inferior parietal cortex	R	40/39	14	40 −56 44	−0.10(0.20)*	−0.22(0.13)*	−0.14(0.16)*	−0.23(0.14)*
Precuneus	R	NA	11	16 −59 41	−0.03(0.21)	−0.15(0.12)*	−0.08(0.18)*	−0.18(0.13)*
*Decreased positive connectivity*								
Medial orbitofrontal cortex	L	10/11	22	−10 44 −10	0.01(0.18)	0.15(0.15)*	0.09(0.17)*	0.21(0.18)*
Inferior orbitofrontal cortex	L	11/48/38	21	−23 14 −22	−0.004(0.14)	0.21(0.20)*	0.08(0.19)*	0.22(0.13)*
*Others* d								
Parahippocampus/Lingual cortex	L	37	12	−28 −43 −6	0.04(0.17)	0.17(0.16)*	0.16(0.21)*	0.04(0.14)
Inferior orbitofrontal cortex/Pars triangularis	L	47/45	11	−46 26 −2	0.01(0.19)	0.13(0.16)*	0.14(0.18)*	0.0040(0.24)

a k = number of voxels in the particular cluster, only cluster-size (k)>10 voxels were listed.

b In Montreal Neurological Institute (MNI) space.

c Mean (standard deviation).

d The changed patterns of functional connectivity were inconsistent between schizophrenic patients and their unaffected parents.

*indicates a significant functional connectivity (p<0.05) with the left amygdala within groups.

The brain imaging results were labeled with the Automated Anatomical Labeling (AAL) software (82) in combination with the Brodmann templates implemented in MRIcroN software (http://www.cabiatl.com/mricro/mricron). SZ, schizophrenic patients; HC1, healthy controls for schizophrenic patients; PA, parents of schizophrenic patients; HC2, healthy controls for parents; BA, Brodmann area; NA, nonapplicable.

#### Right amygdala FC

Relative to the HC1 controls, the schizophrenic group exhibited significantly reduced positive connectivity to the right hippocampus, left precuneus and left fusiform cortex and showed reduced negative connectivity to the right precuneus and right angular cortex. They also showed increased positive connectivity to the right putamen and right caudate nucleus. Increased negative connectivity was observed primarily to the left supplementary motor area in the SZs (Figure S1B, Table S5). Relative to the HC2 controls, the parent group only showed increased positive connectivity in the right transverse temporal gyrus, left inferior frontal gyrus and bilateral insulae (Figure S1B, Table S6). No FC changes in the right amygdala were found in either the schizophrenic patients or their parents (Figure S1C).

## Discussion

The principle finding of our study was the sharing of structural and functional abnormalities of the amygdala in schizophrenic patients and their unaffected parents. As far as we know, this is the first evidence from multimodal imaging that reveals brain structural and resting-state functional abnormalities of the amygdala in schizophrenia families.

### Structural disturbances of the amygdala

Our findings of decreased GMD in schizophrenic patients are consistent with previous studies, which reported GMD changes in the prefrontal and temporal cortices, the amygdala-hippocampal region, and the thalamus [9], [34], [35]. The finding that, compared with their controls the parents showed pronounced GMD reductions mainly in the left amygdala and the left thalamus, is compatible with previous neuroanatomical studies of relatives of schizophrenic patients [36], [37], [38]. Moreover, a shared GMD reduction in the left amygdala occurred in schizophrenic patients and their parents.

Among these findings, the shared structural abnormalities of the amygdalae were especially interesting. Numerous studies have addressed structural disturbances of the amygdala and its role in the pathophysiology of schizophrenia [35], [39], [40], [41], [42], [43]. For example, neuroanatomical abnormalities in the amygdala have been implicated in deficient emotional processing [42], poor verbal memory [44] and psychotic symptoms [45] in schizophrenia. Moreover, other investigations of unaffected relatives of schizophrenia patients have also suggested structural changes in the amygdala-hippocampus region [36], [44], [46], [47]. Because of spatial resolution limitations and for technical reasons, previous ROI-based MRI studies have generally combined the amygdala and hippocampus into a complex, leaving in question the separate involvement of the hippocampus or amygdala. With the development of high resolution MRI and improved methodological approaches, investigating tiny human brain structures has become possible [48], [49]. However, recent voxel-based morphometry studies of relatives of schizophrenic patients have produced mixed findings: a cross-sectional study reported no significant structural changes in the amygdala [50], whereas a longitudinal study found gray matter reductions in the amygdala [38]. Using a high resolution 3T MRI, we obtained fresh evidence of structural changes in the amygdala in both schizophrenic patients and their unaffected relatives. This finding reemphasized previous discoveries of structural disturbances of the amygdala in schizophrenia and their potential as markers for genetic liability for this disease [51].

Unlike the schizophrenic patients in our study, their parents showed no significant gray matter loss in several previously reported regions, such as the hippocampus and cingulate cortex [14], [16], [52]. Variability between samples as well as methodological approaches could be responsible for this inconsistency. In addition, studies of relatives appear to show smaller effects on brain structural changes (e.g. gray matter reductions in the hippocampus) than similar studies in schizophrenia patients [14]. These smaller effects in relatives suggest that proportionally larger samples would be required to obtain the same power as patient studies to detect group differences [18]. Thus, to better understand schizophrenia etiology, greater effort is needed in the search for biological markers that are robustly associated with genetic liability.

### Dysfunctional connectivity of the amygdala

We found that the amygdala showed significantly reduced positive connectivity with the SFC, MPFC, and OFC and significantly reduced negative connectivity with the DLPFC and precuneus in schizophrenic patients (Figure 2B, Table S3). These findings are compatible with previous fMRI studies in which schizophrenic patients showed marked functional anomalies of the amygdala-frontal circuit in the resting state [53] and during a large number of social-emotional tasks [10], [11], [54], [55]. According to Phillips et al. [56], the amygdala, the OFC, and other specific ventral regions constitute the ventral system, which is important for appraisal and identification of emotive stimuli and generation of appropriate emotional responses. The dorsal system, however, consists of the DLPFC, dorsal MPFC and other specific dorsal regions and is employed to maintain the resulting emotional response, including modulating this behavior effectively to meet contextual demands posed by the environment and the individual's internal goals. A reciprocal functional relationship may exist between the two systems, and structural and functional abnormalities within the ‘circuit’ may result in impairments in emotion processing [57]. This concept was supported by recent evidence which indicated that the strength of the RSFC between the amygdala and ventral prefrontal cortex has a significant inverse relationship with aggression in schizophrenia [53]. Hence, we speculate that the altered FCs that we found may be indicators of dysfunctional integration of the ‘circuit’ for emotional processing, a speculation which is consistent with the increasing evidence that schizophrenia is a disorder characterized by dysfunctional integration between brain regions [21], [22], [23].

We found that the left amygdala showed significantly reduced functional connectivity with the left OFC, right DLPFC and right precuneus in schizophrenic patients as well as in their parents (Figure 2C, Table 1). Our data are in line with a recent task-dependent fMRI study in which patients and their unaffected siblings showed less activity in the amygdala following a negative mood induction [13]. Although the exact neurophysiologic mechanism of the regulation of the activity of the resting brain remains unclear, growing evidence indicates that genetic factors may play a role [15], [32], [33], [58], [59] thus paving the way for using resting-state indices as endophenotypes of psychiatric illnesses [60]. In particular, previous studies found that the interregional FCs in schizophrenic patients and first-degree relatives were quite similar during the resting state [15], [33], [58]. Therefore, examining the RSFCs in unaffected relatives of schizophrenic patients may contribute to the identification of schizophrenia endophenotypes. Our overlap result suggests that dysfunctional connectivity of the left amygdala is a potential endophenotype of schizophrenia.

### The co-occurrence of structural and functional abnormalities of the amygdala

Although recent studies have increased our understanding of the relationship between FC and its substrates in schizophrenia [61], [62], most previous work concentrated on the functional correlates of white matter lesions and little work has been done to examine how changes in gray matter may be associated with changes in RSFC. To our knowledge, only one previous study by Lui et al. [29] has addressed this issue. Even though they did not observe an altered functional network, they found that functional networks that involved areas with focal anatomical lesions were associated with clinical symptom severity in schizophrenia. Other researchers have suggested that using neuroimaging measures which are functionally related to each other would be more useful in understanding the pathophysiological pathways [20]. In response to this, previous task-dependent MRI studies have revealed an association between focal gray matter abnormalities and altered functional connectivity in schizophrenia [63], [64]. In our RSFC analysis, we obtained empirical evidence that supports findings obtained by modeling the impact of lesions in the human brain [65], which showed that anatomical lesions may produce altered RSFCs between distant brain regions. Additionally, the striking finding of our study was the similarly changed structural and functional patterns of the amygdala in schizophrenia families. A key point is that co-occurrences of abnormalities in the structure and function of brain regions in schizophrenic patients and their first-degree relatives may suggest a functionally linked set of biological markers associated with genetic disposition toward schizophrenia. We, therefore, speculate that gray matter changes may lead to a systems-level alteration in RSFC and that a genetic basis may underlie the relationship between brain structural and functional features in schizophrenia. Although more studies with increased numbers of subjects are needed, this present work may well provide a basis for further research that will lead to improved comprehension of schizophrenia pathophysiology.

### Issues

Two areas of concern can be raised about our use of parents as a relatives group in this family study of schizophrenia. First, the average age of the parents we sampled differs by nearly 30 years from that of their schizophrenic offspring. Because of age effects, a direct comparison between unaffected parents and their schizophrenic offspring (or the patients' controls) is not reasonable, so the inclusion of the parents necessitated a separate control group. Second, the average lifetime risk for developing schizophrenia is lower in parents (6%) compared with siblings (9%) and offspring (13%) [3]. Therefore, because parents have already passed the age of peak risk for schizophrenia (typically late adolescence or early twenties, see review [66]), their inclusion in this study allowed us to evaluate any underpinnings of schizophrenia that are independent of the disease state. In contrast, investigating younger relatives such as offspring provides an opportunity to identify the neurobiological differences present prior to the typical age of onset of schizophrenia. Younger relatives are likely to be comprised of a mixture of future cases and noncases, whereas parents are unlikely to have any further onset of illness [18].

Although the VBM analysis revealed common GMD changes between the schizophrenia and their parents only in the left amygdala, the right amygdala was also employed as an ROI in our FC analysis. Interestingly, we found no overlap in dysfunctional connectivity between SZ and PA in the right amygdala ROI-driven FC maps. Our data indicates that the left amygdala is implicated to a greater extent than the right amygdala in schizophrenia families. Previous studies have indicated that pathological findings in schizophrenia, such as in vivo neuroanatomical alterations [67], [68] or neurobiological abnormalities [69], are more pronounced in the left amygdala. In addition, neurochemical post-mortem studies of schizophrenia demonstrated that a specific increase in dopamine is found in the left amygdala [70]. A previous family study also revealed that schizophrenic patients and their first-degree relatives shared disturbances in the normal pattern of neuroanatomical asymmetry of the amygdala [71]. Though no firm conclusions can be drawn, the possibility of lateralized pathology in schizophrenia has been proposed [72]. Interactions between cerebral asymmetry, gender, and brain development introduce further complexity to the issue [73]. Clarifying how neuropathological changes interact with cerebral lateralization thus requires additional, appropriately designed studies of schizophrenia.

### Limitations and Future Work

There are two possible limitations in evaluating our results. First, the effect of antipsychotics may be a possible confounding factor in our findings. However, a study in first-episode, antipsychotic-naïve schizophrenic patients demonstrated that amygdala abnormalities exist in the early phases of schizophrenia, prior to and in the absence of antipsychotic treatment [40]. Moreover, GMD reductions and altered FC were found in parents of schizophrenic patients that did not have a history of antipsychotic medication. These findings would imply that antipsychotic medications do not primarily account for the structural and functional abnormalities in the amygdala of patients. Second, the current study did not establish a quantitative relationship with respect to the neuroimaging measures between the patients and their parents. In a previous study, Honea et al. [74] used an intraclass correlation to explore significant familial changes in brain regions in schizophrenic patients and their unaffected siblings. However, our study design was special in that both biological parents of the patients were recruited. Calculating intraclass correlations between schizophrenia probands and their parents is unsuitable because the number of parents was almost twice than that of schizophrenia probands. Recently, sophisticated algorithms have been developed that can perform family-based association tests for use with quantitative traits [75], [76], [77]. These methods may allow investigators to find intermediate phenotypes and common gene variants that could indicate schizophrenia susceptibility. But these tests generally require a large sample and may impose a financial burden on researchers using MRI studies. Hopefully, future experiments using some combined methodological approach to heritability and a larger sample may address the unsolved question of the genetic aspects of schizophrenia as evidenced by common changes in both schizophrenic patients and their parents.

### Conclusions

In the present study, we found co-occurrences of structural and functional abnormalities of the amygdala in both schizophrenic patients and their parents. Although the neuropathology of schizophrenia remains obscure, our findings suggest that the structural and functional abnormalities of the amygdala in schizophrenia have a genetic basis. These findings can help to accelerate the identification of ideal endophenotypes that will better characterize the complex disorder of schizophrenia.

## Materials and Methods

### Ethics statement

The study was approved by the Medical Research Ethics Committee of the Institute of Mental Health, Peking University. All participants were given detailed verbal and written information regarding the purpose and procedures of the study. Written consents were obtained from the patients and/or their parents, and all healthy participants enrolled in this study, among which 2 out-patients with mild symptoms signed the written consents all by themselves.

### Subjects

One hundred and forty-seven subjects in the Chinese Han population, including 33 schizophrenic patients (SZ), 55 of their unaffected biological parents (PA), 30 young healthy controls for the SZ (HC1) and 29 old healthy controls for the PA (HC2), took part in this study. The SZ and PA groups were recruited through the Institute of Mental Health, Peking University. Exclusion criteria were electroconvulsive therapy within 6 months, a history of serious medical illness, and excessive head motion in the subsequent data analysis. Three patients were excluded based on these criteria. Two trained and experienced psychiatrists ensured that the patients satisfied the ICD-10 diagnostic criteria for research for schizophrenia with paranoid subtype [78]. At the time of MR scanning, all patients were receiving antipsychotic medications, for which the dosages were converted into chlorpromazine equivalents [79], [80], [81]. The evaluation of disease severity and psychopathology was assessed by experienced psychiatrists using the Positive and Negative Syndrome Scale (PANSS) [82]. Age- and gender-matched controls, none of whom reported any first- or second-degree relatives with schizophrenia, were recruited from the local community. Psychiatrists conducted unstructured interviews with the unaffected parents and healthy controls to exclude individuals with a history of head injury, neurological disease or severe mental disease. All participants (patients, parents and both control groups) were right-handed, as assessed by the Edinburgh Handedness Inventory [83], and had no intracranial pathology, history of head injury, neurological disorder, alcohol or substance abuse. Demographic and clinical characteristics of the patients, their parents and controls are summarized in Table 2.

**Table 2:** Demographic and clinical characteristics of the patients, their parents, and the normal controls.

Variable	SZ(n = 30)	HC1(n = 30)	*p*	PA(n = 55)	HC2(n = 29)	*p*
Gender (male/female)	17/13	18/12	0.79^a^	27/28	14/15	0.94^a^
Age, years	22.63(3.76)^b^	22.77(3.34)	0.89^c^	50.31(5.10)	51.79(5.58)	0.22^c^
Education, years	13.67(1.95)	14.30(1.99)	0.22^c^	13.25(2.89)	13.10(2.64)	0.82^c^
Onset, years	19.43(3.21)					
Duration of illness, months	39(33.27)					
Medication dose, mg^d^	407.67(240.57)					
PANSS positive score	19.37(4.60)					
PANSS negative score	16.13(4.65)					
PANSS total score	67.27(11.81)					

a Pearson Chi-square test.

b Mean (standard deviation).

c Two sample *t*-test.

d Chlorpromazine-equivalent dose.

SZ, schizophrenic patients; HC1, healthy controls for schizophrenic patients; PA, parents of schizophrenic patients; HC2, healthy controls for parents; PANSS, Positive and Negative Syndrome Scale.

### Data acquisition

MRI scans were obtained at the Department of Radiology, The Third Hospital, Peking University, with a 3.0-Tesla Magnetom Trio (Siemens Medical System, Erlangen, Germany) using foam pads to reduce head motion and scanner noise. Three-dimensional T1-weighted images were acquired in a sagittal orientation employing a 3D-MPRAGE sequence with the following parameters: time repetition (TR) = 2350 ms, time echo (TE) = 3.44 ms, flip angle = 7°, matrix size = 256×256, field of view (FOV) = 256×256 mm^2^, 192 sagittal slices, slice thickness = 1 mm, acquisition voxel size = 1.0×1.0×1.5 mm^3^, total acquisition time  =  363 s. After collecting structural MRI scans, resting-state fMRI scans were acquired. During resting-state scanning, the subjects were instructed to lie still with their eyes closed, not to think systematically, and not to fall asleep. The 7-minute resting-state scan was comprised of 210 contiguous echo planar imaging whole brain functional volumes with the following parameters: TR = 2000 ms, TE = 30 ms, flip angle = 90°, matrix size = 64×64, FOV = 220×220 mm^2^, 30 axial slices, thickness/slice gap = 4/0.8 mm, acquisition voxel size = 3.438×3.438×4.8 mm^3^.

### Structural image processing

To identify gray matter alternations in schizophrenia families, VBM was performed using the VBM5 toolbox (http://dbm.neuro.uni-jena.de) with the SPM5 software (http://www.fil.ion.ucl.ac.uk/spm). A detailed description of the processing procedure of the VBM5 toolbox can be found elsewhere [84]. This procedure generally yields modulated and unmodulated tissue images. Based on previous published family studies of schizophrenia [85], [86], [87], and our experience [88], we only adapted unmodulated data for the GMD analysis in the present study. The resulting gray matter images (voxel size 1×1×1 mm^3^) were smoothed with a 6-mm full width-half maximum (FWHM) Gaussian kernel.

### Functional connectivity analysis

#### Regions of interest definition

Based on an explorative VBM analysis across the whole brain, GMD changes in the left amygdala were found in common between schizophrenic patients and their unaffected parents. We were also interested in whether the patients and their parents would show similar abnormalities in the RSFC of the amygdala. Because the amygdala is a bilateral brain structure, we still concerned the functional integration of the right amygdala during the resting state in schizophrenia families. To this end, we performed a FC analysis using the bilateral amygdalae. Regions with gray matter loss could reasonably be directly defined as seed areas for functional connectivity analysis. However, directly defining regions this way may result in less consistent spatial localization of FC differences from sample to sample, because slight changes in the positioning of the seed areas could significantly change the functional network [89]. Thus, we defined the replicable amygdala ROIs based on structural atlases from stereotaxic probabilistic maps (http://www.fmrib.ox.ac.uk/fsl/fslview/atlas-descriptions.html). The advantage of such probabilistic maps is that they provide information about the location and inter-individual variability of brain areas in standard reference space. A modest threshold of 50% was applied. This means that only voxels with at least a 50% probability of belonging to the amygdala were included (left: 1939 mm^3^; right: 2276 mm^3^).

#### Functional imaging processing

The fMRI data preprocessing procedures were performed using the Resting-State fMRI Data Analysis Toolkit (REST, http://www.restfmri.net) and included: 1) removal of the first 10 scans, 2) slice time correction, 3) head motion correction, 4) spatial normalization via structural segmentation carried out by VBM5 (fMRI data were subsampled to 3×3×3 mm^3^), 5) spatial smoothing with a 6-mm FWHM Gaussian kernel, 6) linear detrending and temporal bandpass filtering (0.01–0.08 Hz). Based on the head motions recorded in each fMRI run, three patients were excluded due to excessive movements; all the rest of the participants (n = 144) had less than 3 mm maximum displacement and less than 3° of angular rotation. Because FC analysis is sensitive to gross head motion effects, we further characterized the peak displacements and mean displacements as measures of head motion for each subject following the method described elsewhere [90], [91]. A two-sample *t*-test revealed no significant between-group differences (Table S7).

#### Functional connectivity calculation

For the defined ROIs, each voxel's time series was extracted and weighted by its probability of being in the amygdala. Then an averaged time course within the left and right amygdalae, separately, was calculated as the reference time series. A partial correlation analysis was conducted between the ROI reference and the rest of the brain in a voxel-wise manner [92]. Nine nuisance covariates, including 3 averaged time courses extracted from predefined masks of the whole brain, white matter and cerebrospinal fluid, and six head motion time courses, were used to regress out confounding factors. This analysis produced individual subject-level maps of all positively- and negatively-correlated voxels for the ROI seed. Partial correlation coefficients were then calculated and converted to *z*-values using Fisher's *z*-transformation [91].

### Statistical Analysis

#### GMD differences

To test whether regional gray matter was significantly altered in the SZ and PA groups, voxel-by-voxel based comparisons of GMD were computed to determine differences between groups using two-sample *t*-tests, contrasting SZ with HC1, and PA with HC2. For each contrast, an uncorrected *p*<0.01 at the voxel level and a minimum cluster size of 979 mm^3^ were utilized to correct for multiple comparisons. This yielded a corrected threshold of *p*<0.05, determined by using AlphaSim with 5000 Monte Carlo stimulations (see program AlphaSim by D. Ward in AFNI software, http://afni.nimh.nih.gov/pub/dist/doc/manual/AlphaSim.pdf).

#### FC differences

To identify the within-group RSFC, individual *z*-maps were entered into one-sample *t*-tests in a voxel-wise manner to determine the brain regions showing significantly positive or negative correlation with the ROI in each group. An uncorrected *p*<0.01 at the voxel level and a cluster size of at least 1107 mm^3^ were utilized to correct for multiple comparisons and yielded a corrected threshold of *p*<0.05 as determined using AlphaSim. Then the within-group connectivity maps that survived the given threshold were used to generate a binary mask which ensured that the *z*-values of connectivity were significantly different from zero in at least one group. To identify significant FC differences between groups, voxel-by-voxel two-sample *t*-tests (within the binary mask) were performed on individual RSFC maps to determine differences between groups, contrasting SZ with HC1, and PA with HC2. For the left amygdala ROI, a combined threshold of contrast maps was set at an uncorrected *p*<0.05 at the voxel level and a minimum cluster size (2862 mm^3^ for SZ versus HC1 and 3078 mm^3^ for PA versus HC2). These combined thresholds of height and extent correspond to a corrected threshold of *p*<0.05, determined using AlphaSim. For the right amygdala ROI, a combined threshold of contrast maps was set at an uncorrected *p*<0.05 at the voxel level and a minimum cluster size (2754 mm^3^ for SZ versus HC1 and 3051 mm^3^ for PA versus HC2). These combined thresholds of height and extent correspond to a corrected threshold of *p*<0.05, determined using AlphaSim.

### Overlap mapping

To enable the visualization of the areas in which we found overlap between the groups, separate surface renderings of the two thresholded between-group maps (*p*<0.05, corrected) were used for the VBM and FC analyses. This processing allowed us to identify the neuropathological features shared by schizophrenic patients and their first-degree relatives. Geometric centers of the overlap regions were determined in MNI space.

## Supporting Information

Figure S1 **Surface renderings representing the results from the analysis of functional MRI data.** Statistical parametric maps of functional connectivity analysis of the right amygdala are superimposed on the population, landmarked-and surface-based (PALS) atlas of the human cerebral cortex [93] using CARET software (http://brainvis.wustl.edu). (A) Within-group functional connectivity patterns. Cool color and warm color, respectively, are used to indicate regions with significantly negative connectivity and significantly positive connectivity (*p*<0.05, corrected) with the right amygdala. (B) Between-group functional connectivity patterns. When compared with respective counterparts, regions with significantly (*p*<0.05, corrected) altered function connectivity with the right amygdala in schizophrenic patients and their parents are shown. Color bar indicates the *z*-value. (C) An overlapping surface rendering of the between-group statistical parametric maps (SZ versus HC1, light green; PA versus HC2, light blue). The absence of yellow areas indicates that no overlap regions were found. SZ, schizophrenic patients; HC1, healthy controls for schizophrenic patients; PA, parents of schizophrenic patients; HC2, healthy controls for parents.
(TIF)

Table S1 Brain regions with decreased gray matter density in schizophrenic patients.
(DOC)

Table S2 Brain regions with decreased gray matter density in the parents of patients.
(DOC)

Table S3 Brain regions which showed significant differences in the functional connectivity of the left amygdala between schizophrenic patients and healthy controls for patients.
(DOC)

Table S4 Brain regions which showed significant differences in the functional connectivity of the left amygdala between the parents of patients and healthy controls for parents.
(DOC)

Table S5 Brain regions which showed significant differences in the functional connectivity of the right amygdala between schizophrenic patients and healthy controls for patients.
(DOC)

Table S6 Brain regions which showed significant differences in the functional connectivity of the right amygdala between the parents of patients and healthy controls for parents.
(DOC)

Table S7 Head movement analysis.
(DOC)
