# Supplementary figures and images for: Convergent Evidence from Multimodal Imaging Reveals Amygdala Abnormalities in Schizophrenic Patients and Their First-Degree Relatives
Source: PLoS One. 2011 Dec 8;6(12):e28794. doi: 10.1371/journal.pone.0028794 (PMC3234284; doi:10.1371/journal.pone.0028794)

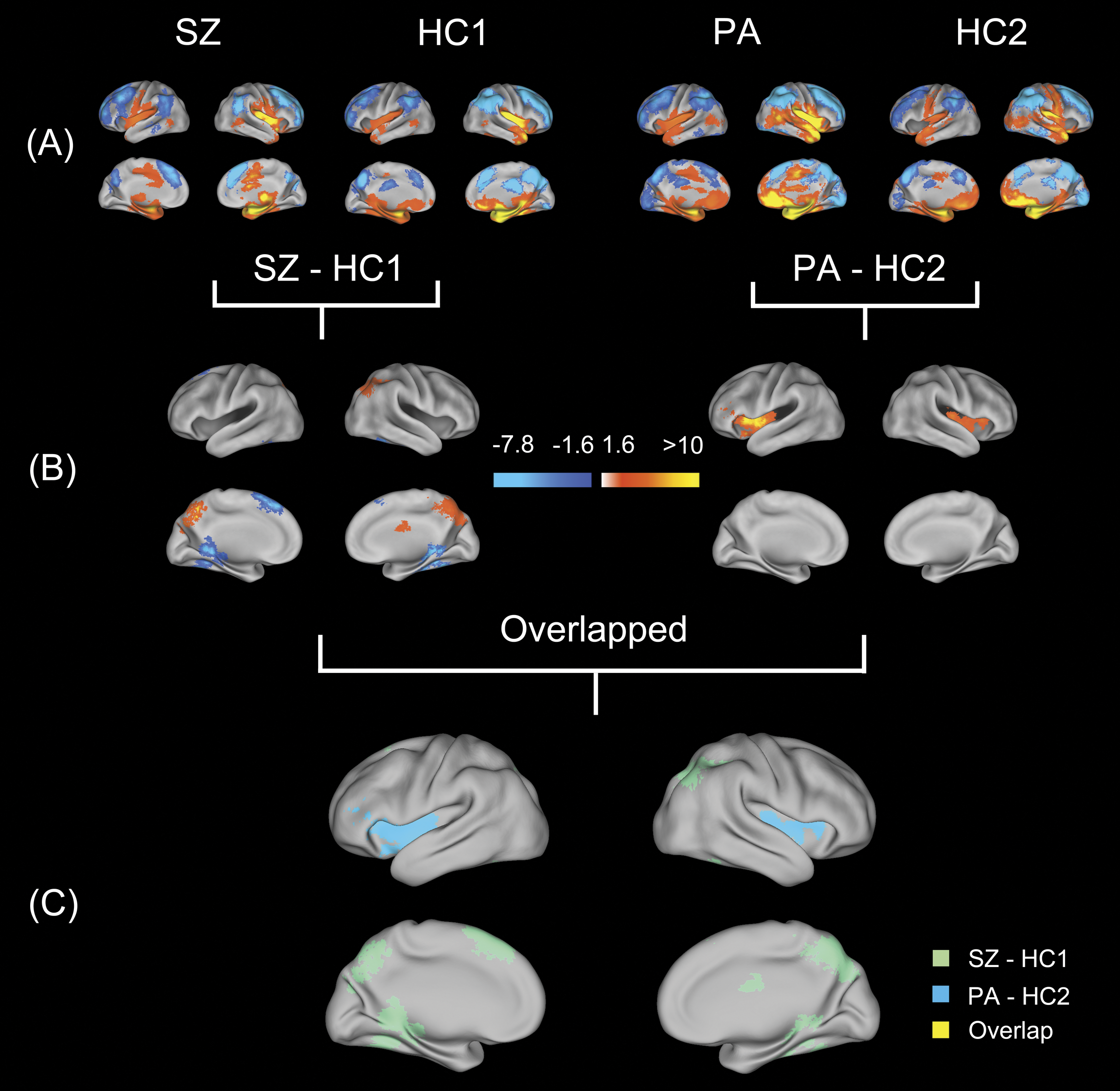

Supplement: Figure S1 — Surface renderings representing the results from the analysis of functional MRI data. Statistical parametric maps of functional connectivity analysis of the right amygdala are superimposed on the population, landmarked-and surface-based (PALS) atlas of the human cerebral cortex [93] using CARET software (http://brainvis.wustl.edu). (A) Within-group functional connectivity patterns. Cool color and warm color, respectively, are used to indicate regions with significantly negative connectivity and significantly positive connectivity (p<0.05, corrected) with the right amygdala. (B) Between-group functional connectivity patterns. When compared with respective counterparts, regions with significantly (p<0.05, corrected) altered function connectivity with the right amygdala in schizophrenic patients and their parents are shown. Color bar indicates the z-value. (C) An overlapping surface rendering of the between-group statistical parametric maps (SZ versus HC1, light green; PA versus HC2, light blue). The absence of yellow areas indicates that no overlap regions were found. SZ, schizophrenic patients; HC1, healthy controls for schizophrenic patients; PA, parents of schizophrenic patients; HC2, healthy controls for parents. (TIF) [file pone.0028794.s001.tif]
