# Supplementary material for: Convergent Evidence from Multimodal Imaging Reveals Amygdala Abnormalities in Schizophrenic Patients and Their First-Degree Relatives
Source: PLoS One. 2011 Dec 8;6(12):e28794. doi: 10.1371/journal.pone.0028794 (PMC3234284; doi:10.1371/journal.pone.0028794)
Supplement: Table S2 — Brain regions with decreased gray matter density in the parents of patients. (DOC) [file pone.0028794.s003.doc]

**Table S2.** Brain regions with decreased gray matter density in the parents of patients

| **Regions** | **Cluster-size (k)** | ***t*-scores of peak voxel** | **Coordinates** |
| --- | --- | --- | --- |
| Amyglada_L | 1079 | 4.52 | -26 4 -14 |
| Olfactory_L |  | 2.93 | -13 12 -13 |
| Frontal_Superior_Orb_R | 1032 | 4.43 | 20 52 -10 |
| Frontal_Med_Orb_R |  | 3.86 | 8 60 -13 |
| Frontal_Sup_Orb_R |  | 3.01 | 21 57 2 |
| Insula_L | 1264 | 4.05 | -35 -1 8 |
| Pallidum_L | 1612 | 3.64 | -22 -5 -2 |
| Thalamus_L |  | 3.58 | -16 -7 6 |
| Thalamus_L |  | 3.46 | -21 -20 10 |

Note: The brain imaging results reported in the supplementary tables were labeled with the Automated Anatomical Labeling (AAL) software (4). Anatomical labels of the peak coordinates were reported in Montreal Neurological Institute (MNI) space. L = left; R = right; Inf = inferior; Post = posterior; Mid = middle; Sup = superior; Med = medial; Tri = triangle; Orb = orbital; Oper = opercular; k = number of voxels in the particular cluster.
