# Supplementary material for: Convergent Evidence from Multimodal Imaging Reveals Amygdala Abnormalities in Schizophrenic Patients and Their First-Degree Relatives
Source: PLoS One. 2011 Dec 8;6(12):e28794. doi: 10.1371/journal.pone.0028794 (PMC3234284; doi:10.1371/journal.pone.0028794)
Supplement: Table S5 — Brain regions which showed significant differences in the functional connectivity of the right amygdala between schizophrenic patients and healthy controls for patients. (DOC) [file pone.0028794.s006.doc]

**Table S5.** Brain regions which showed significant differences in the functional connectivity of the right amygdala between schizophrenic patients and healthy controls for patients

| **Regions** | **Cluster-sizes (k)** | ***t*-scores of peak voxel** | **Coordinates** | **SZ** | **HC1** | **FC changes in SZ** |
| --- | --- | --- | --- | --- | --- | --- |
| SZ - HC1 > 0 |  |  |  |  |  |  |
| Putamen_R | 215 | 3.89 | 27 3 9 | + | + | Increased positive connectivity |
| Caudate_R |  | 3.4 | 15 12 6 | + | N.S. | Increased positive connectivity |
| Cuneus_R | 310 | 3.65 | 12 -75 36 | N.S. | - | Decreased negative connectivity |
| Precuneus_R |  | 3.33 | 12 -69 60 | N.S. | - | Decreased negative connectivity |
| Parietal_Superior_L |  | 3.02 | -15 -60 48 | N.S. | - | Decreased negative connectivity |
| Angular_R | 183 | 3.41 | 36 -66 48 | N.S. | - | Decreased negative connectivity |
| Angular_R |  | 3.28 | 36 -72 42 | N.S. | - | Decreased negative connectivity |
| Angular_R |  | 3.11 | 33 -57 42 | N.S. | - | Decreased negative connectivity |
| SZ - HC1 < 0 |  |  |  |  |  |  |
| Precuneus_L | 195 | -4.02 | -15 -48 3 | N.S. | + | Decreased positive connectivity |
| Fusiform_L |  | -3.42 | -33 -60 -15 | N.S. | + | Decreased positive connectivity |
| Frontal_Superior_Medial_L | 121 | -3.48 | -3 33 54 | - | N.S. | Increased negative connectivity |
| Supplementary_Motor_Area_L |  | -3.31 | -6 15 69 | - | N.S. | Increased negative connectivity |
| Supplementary_Motor_Area_L |  | -3.22 | 0 24 60 | - | N.S. | Increased negative connectivity |
| Hippocampus_R | 345 | -3.16 | 30 -33 -6 | N.S. | + | Decreased positive connectivity |
| Hippocampus_R |  | -2.86 | 33 -36 3 | N.S. | + | Decreased positive connectivity |

Note: The brain imaging results reported in the supplementary tables were labeled with the Automated Anatomical Labeling (AAL) software (4). Anatomical labels of the peak coordinates were reported in Montreal Neurological Institute (MNI) space. L = left; R = right; Inf = inferior; Post = posterior; Mid = middle; Sup = superior; Med = medial; Tri = triangle; Orb = orbital; Oper = opercular; k = number of voxels in the particular cluster; SZ, schizophrenic patients; HC1, healthy controls for patients; FC, functional connectivity; + indicates positive connectivity with the ROI within groups; - indicates negative connectivity with the ROI within groups; N.S. indicates no significant functional connectivity with the ROI within groups.
