# Supplementary material for: Convergent Evidence from Multimodal Imaging Reveals Amygdala Abnormalities in Schizophrenic Patients and Their First-Degree Relatives
Source: PLoS One. 2011 Dec 8;6(12):e28794. doi: 10.1371/journal.pone.0028794 (PMC3234284; doi:10.1371/journal.pone.0028794)
Supplement: Table S6 — Brain regions which showed significant differences in the functional connectivity of the right amygdala between the parents of patients and healthy controls for parents. (DOC) [file pone.0028794.s007.doc]

**Table S6.** Brain regions which showed significant differences in the functional connectivity of the right amygdala between the parents of patients and healthy controls for parents

| **Regions** | **Cluster-sizes (k)** | ***t*-scores of peak voxel** | **Coordinates** | **PA** | **HC2** | **FC changes in PA** |
| --- | --- | --- | --- | --- | --- | --- |
| PA - HC2 > 0 |  |  |  |  |  |  |
| Heschl_R | 194 | 4.77 | 42 -18 12 | + | N.S. | Increased positive connectivity |
| Insula_R |  | 3.46 | 36 21 6 | + | N.S. | Increased positive connectivity |
| Insula_R |  | 3.26 | 45 -3 -3 | + | N.S. | Increased positive connectivity |
| Temporal_Superior_L | 344 | 3.74 | -48 -6 -6 | + | N.S. | Increased positive connectivity |
| Insula_L |  | 3.57 | -36 18 0 | + | N.S. | Increased positive connectivity |
| Frontal_Inferior_Triangle_L | 113 | 3.39 | -45 24 0 | + | N.S. | Increased positive connectivity |

Note: The brain imaging results reported in the supplementary tables were labeled with the Automated Anatomical Labeling (AAL) software (4). Anatomical labels of the peak coordinates were reported in Montreal Neurological Institute (MNI) space. L = left; R = right; Inf = inferior; Post = posterior; Mid = middle; Sup = superior; Med = medial; Tri = triangle; Orb = orbital; Oper = opercular; k = number of voxels in the particular cluster; PA, parents of schizophrenic patients; HC2, healthy controls for parents; FC, functional connectivity; + indicates positive connectivity with the ROI within groups; - indicates negative connectivity with the ROI within groups; N.S. indicates no significant functional connectivity with the ROI within groups.
