# Supplementary material for: Convergent Evidence from Multimodal Imaging Reveals Amygdala Abnormalities in Schizophrenic Patients and Their First-Degree Relatives
Source: PLoS One. 2011 Dec 8;6(12):e28794. doi: 10.1371/journal.pone.0028794 (PMC3234284; doi:10.1371/journal.pone.0028794)
Supplement: Table S7 — Head movement analysis. (DOC) [file pone.0028794.s008.doc]

**Table S7.** Head movement analysis

|  | **SZ** | **HC1** | ***p*** | **PA** | **HC2** | ***p*** |
| --- | --- | --- | --- | --- | --- | --- |
|  | Mean ± SD | Mean ± SD |  | Mean ± SD | Mean ± SD |  |
| Mean displacement, mm | 0.31 ± 0.22 | 0.27 ± 0.23 | 0.50^a^ | 0.34 ± 0.22 | 0.35 ± 0.22 | 0.72 ^a^ |
| Peak displacement, mm | 0.75 ± 0.61 | 0.63 ± 0.52 | 0.45 ^a^ | 0.78 ± 0.52 | 0.83 ± 0.59 | 0.69 ^a^ |

^a^ Two sample t-test.

SZ, schizophrenic patients; HC1, healthy controls for patients; PA, parents of schizophrenic patients; HC2, healthy controls for parents; SD, standard deviation.
